# An ERP-study of brand and no-name products

**DOI:** 10.1186/1471-2202-14-149

**Published:** 2013-11-23

**Authors:** Anika Thomas, Anke Hammer, Gabriele Beibst, Thomas F Münte

**Affiliations:** 1 Department of Neuropsychology, Otto-von-Guericke-University Magdeburg, Magdeburg, Germany; 2 Department of Business Administration, University of Applied Sciences Jena, Jena, Germany; 3 Department of Neurology, University of Lübeck, Ratzeburger Allee 160, Lübeck 23538, Germany; 4 Department of Psychiatry, University of Erlangen, Erlangen, Germany

**Keywords:** Go/Nogo, Event-related potentials, Brands, Neuromarketing, Implicit associations, Late positive component, Lateralized readiness potential

## Abstract

**Background:**

Brands create product personalities that are thought to affect consumer decisions. Here we assessed, using the Go/No-go Association Task (GNAT) from social psychology, whether brands as opposed to no-name products are associated with implicit positive attitudes. Healthy young German participants viewed series of photos of cosmetics and food items (half of them brands) intermixed with positive and negative words. In any given run, one category of goods (e.g., cosmetics) and one kind of words (e.g., positive) had to be responded to, whereas responses had to be withheld for the other categories. Event-related brain potentials were recorded during the task.

**Results:**

Unexpectedly, there were no response-time differences between congruent (brand and positive words) and incongruent (brand and negative words) pairings but ERPs showed differences as a function of congruency in the 600–750 ms time-window hinting at the existence of implicit attitudes towards brand and no-name stimuli. This finding deserves further investigation in future studies. Moreover, the amplitude of the late positive component (LPC) was found to be enhanced for brand as opposed to no-name stimuli.

**Conclusions:**

Congruency effects suggest that ERPs are sensitive to implicit attitudes. Moreover, the results for the LPC imply that pictures of brand products are more arousing than those of no-name products, which may ultimately contribute to consumer decisions.

## Background

A brand is the personality that identifies a product. Brands like Coca Cola, Ford, or Chanel are deeply embedded in our lives, and companies struggle hard to develop their brands and to provide a unique selling proposition [1]. In most markets, there are competitors selling no-name products which try to gain a share of the business. A central question in marketing research is therefore to what extent positive attitudes towards brands contribute to consumer decisions. Interview statements and verbal self-reports may provide some information [2] but they are notoriously insensitive with regard to the consumer’s decision [3-5]. Moreover, they are insensitive to implicit associations that are linked to unconscious automatic attitudes [6,7]. Brands are thought to implicitly engage specific positive associations (e.g., quality, value, youth, strength, speed, etc.) which are not triggered by no-name products. Such implicit associations may be critical for the consumer’s decision to buy [8-11].

Neuroimaging studies have demonstrated activations of reward related structures such as the striatum and the dorsolateral prefrontal cortex in response to stimuli representing brand products [12-16]. Thus, brands seem to have an implicitly rewarding property. Moreover, Schaefer [16] pointed out that brands, in particular luxury brands, may also be used to mark the social status of the owner and indeed logos of luxury brands were associated with brain activity in the anterior medial prefrontal cortex, a region known to be associated with self-centered cognitions [17].

Another way to assess the presence of implicit attitudes has been proposed by Nosek and Banaji [18] who devised the Go/No-go Association Task (GNAT). This test, disguised as a choice reaction time task, can be used to measure associations between categories (e.g., faces of elderly or young people - assuming that elderly faces are associated with negative attitudes) and either pole of an evaluative dimension (e.g., positive or negative words). Words and category stimuli appear in different trials and randomly intermixed. In any given block of the experiment, participants are required to make a Go-response (button press) to one of the categories under study (e.g., faces of young people) and to one kind of words (e.g., positive words) while withholding responses (Nogo) to the other stimulus types (here: elderly faces, negative words). Category and word stimuli are combined differently for consecutive blocks of the experiment. Go-responses to incongruent pairings (e.g., press for elderly people or positive words) typically result in slower response-times (RTs) as compared to congruent pairings (e.g. elderly people and negative words) showing that the average participant in such an experiment harbors implicit negative attitudes towards the elderly. Differences in response times (RTs) can thus be taken as an index of the strength of an individual’s automatic association, with a bigger difference indicating a stronger implicit attitude. The GNAT and the closely related dual-response Implicit Association Test (IAT) [19] have successfully been used to investigate implicit attitudes towards race, gender and body-weight [18,20] as well as consumer goods [21-24]. With the specific aim to investigate whether implicit or explicit attitudes towards brand and no-name products influence the actual choice of consumers, Friese et al. [10] tested explicit preferences on a 7-point Likert scale and implicit preferences using the IAT. Participants were then given the choice between brand and no-name products. Participants whose explicit and implicit preferences regarding no-name and brand were incongruent more often chose the implicitly preferred brand over the explicitly preferred one when choices were made under time pressure. When no time–pressure was present, the opposite pattern emerged. Thus, implicit attitudes towards consumer products might influence consumer decisions under certain circumstances. A similar finding was recently reported by Beattie and Sale [25] who investigated consumer behavior with regard to high and low carbon dioxide products. While explicit measures did not differentiate the choice of high/low carbon products, the implicit measure did. Again, time pressure was a significant factor. Because of such findings, the importance of implicit measures in consumer research has been stressed recently [9,11].

In previous work [26,27] we have adapted the GNAT paradigm to be used with event-related potentials (ERPs) taking advantage of the fact that studies in various cognitive domains have uncovered robust ERP findings that can be used as chronometric indices for the decision processes leading to the Go or Nogo response. The GNAT lends itself to electrophysiological studies much better than the IAT as it has the advantage to yield two components that can be used to describe the relative timing of information access, the N200 and the lateralized readiness potential (LRP). This lateralized part of the readiness potential has been used as an index for specific response preparation [28] and can be isolated by a double subtraction technique [29-31]. Importantly, the resulting LRP can be obtained for trials requiring a “go”-response, as well as for trials requiring a “nogo” response. In the latter, temporary development of a negative LRP may indicate that some information was present favoring a go-response. Thus, this method allows to derive critical information about the absolute and relative timing of information access even in the absence of overt responses [32-36].

A number of electrophysiological studies have directly compared Nogo and Go-trials and have shown that the stimulus-locked ERP in Nogo-trials is characterized by a large negativity of about 1–4 μV in size occurring with task dependent onset latencies over the fronto-central scalp [37-40]. This frontal “N200” has been linked to inhibitory processes [41-44].

**Figure 1:**
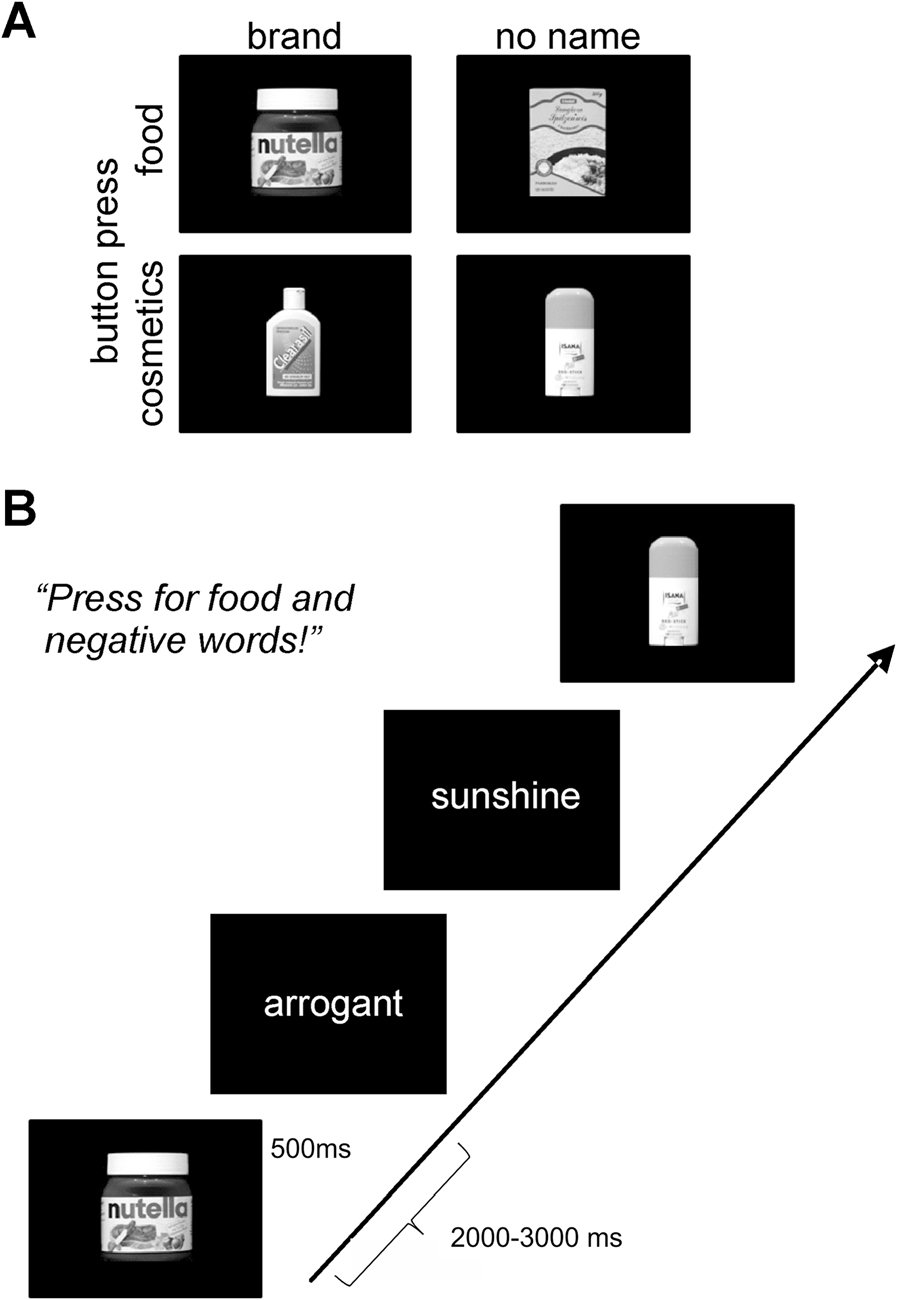
**Paradigm for the elicitation of implicit attitudes. A**: Illustration of the 4 different classes of stimuli. Participants were asked to press (go) or not press (nogo) a response button according to whether or not an item belonged to the cosmetics or food category. Of both categories, half belonged to major German brands whereas the other half represented no-name products. **B**: Pictures of food and cosmetic items were presented in random order intermixed with words with positive or negative valence. In any given run, the participant had to press for one class of picture items (e.g., food) and one class of words (e.g., negative). In the actual experiment, color images were used.

In the present investigation, we combined the GNAT-paradigm with the recording of event-related potentials, thus putting us in the position to assess both, behavioural and neural correlates of implicit associations towards brands (see Figure 1 for an illustration of the experimental set-up). As in similar studies from our lab addressing other topics [26,27,45,46], instructions paired products (brands as compared to no-name-products) from one of two taxonomic categories (cosmetics or food) with either pole of an evaluative dimension (positive or negative words), for example: press for cosmetics or positive words, do not press for food items or negative words. We assumed that brands give rise to implicit positive associations, whereas no-name-products were thought to be linked to implicit negative associations. Participants focused on the decision for words (positive vs. negative) and product categories (food vs. cosmetics) and were naïve to the fact that the experiment addressed the differential processing of brands and no-name-products. Each run required a Go response to one of the taxonomic categories and one evaluative dimension.

We hypothesized that implicit attitudes would be reflected by response latencies for the product decisions such that brands should be responded to faster in blocks that paired the product category of the brand with positive words, whereas response latencies for no-name-products were expected to be faster in blocks that paired the product categories with negative words.

In an unpublished companion study, using fMRI, we found a behavioural effect of product status (brand vs. no-name) on the response latency such that congruent pairings (brand and positive words; no-name products and negative words) led to faster reaction times compared to incongruent pairings. Moreover, comparison of fMRI activations in incongruent and congruent trials revealed significant differences in several brain areas suggesting that brands are implicitly associated with positive attitudes.

Thus, we hypothesized that a similar behavioural pattern should also emerge in the present study. Moreover, similar to our GNAT study assessing implicit attitudes towards fruits and insects [26] and old and young people [27], we expected a latency difference of the N200 component as a function of congruency (longer latency for incongruent trials) which would enable us to locate the effect of brands in time. Likewise, with regard to the lateralized readiness potential, we hypothesized that for congruent blocks the onset of the LRP should be earlier than in incongruent blocks [45]. While in light of our previous work we formulated our hypotheses with regard to the N200 and LRP components, we were also interested in any differences between ERPs recorded in incongruent and congruent conditions, as these could be taken as an indicator of the presence of implicit associations.

Finally, in view of numerous fMRI studies addressing the basic neurophysiological effects of brands [12,14,15,47], we examined general ERP differences between brand and no-name products.

## Results

### Behavioral results

Responses to brand products (congruent = 670 ms, SD = 196; incongruent = 668 ms, SD = 189) were faster than those to no-name products (congruent = 694 ms, SD = 202; incongruent = 694 ms, SD = 204; F(1,15) = 24.56, p < .001). The main effects of Congruency (F(1,15) = 0.07) and Response hand (F(1,15) = 1.47) and the interaction effects (Product x Congruency, F(1,15) = 0.01; Product x Hand, F(1,15) = 0.93; Congruency x Hand, F(1,15) = 1.87; Product x Congruency x Hand, F(1,15) = 1.24) did not reach significance. Response latencies to negative (758 ms, SD = 190) and positive words (773 ms, SD = 197) were statistically indistinguishable (T(15) = 0.86, n.s.).

With regard to errors, only a low error rate was observed for the picture trials (0.94% in Go trials and 5% in Nogo trials). For the Go trials, statistical analysis revealed neither a main effect of Product (F(1,15) = 2.1, p = .17), a main effect of Congruency (F(1,15) = .03, p = .86) nor a significant Product x Congruency interaction (F(1,15) = .12, p = .73). A similar pattern was observed for Nogo-trials (Product, F(1,15) = .12, p = .73; Congruency, F(1,15) = 1.07, p = .32; Product x Congruency, F(1,15) = 1.3, p = .27). Word trials were similarly associated with a low error rate (3.06% in Go trials, 5.0% in Nogo trials).

### Electrophysiology

#### Stimulus-locked ERPs

**Figure 2:**
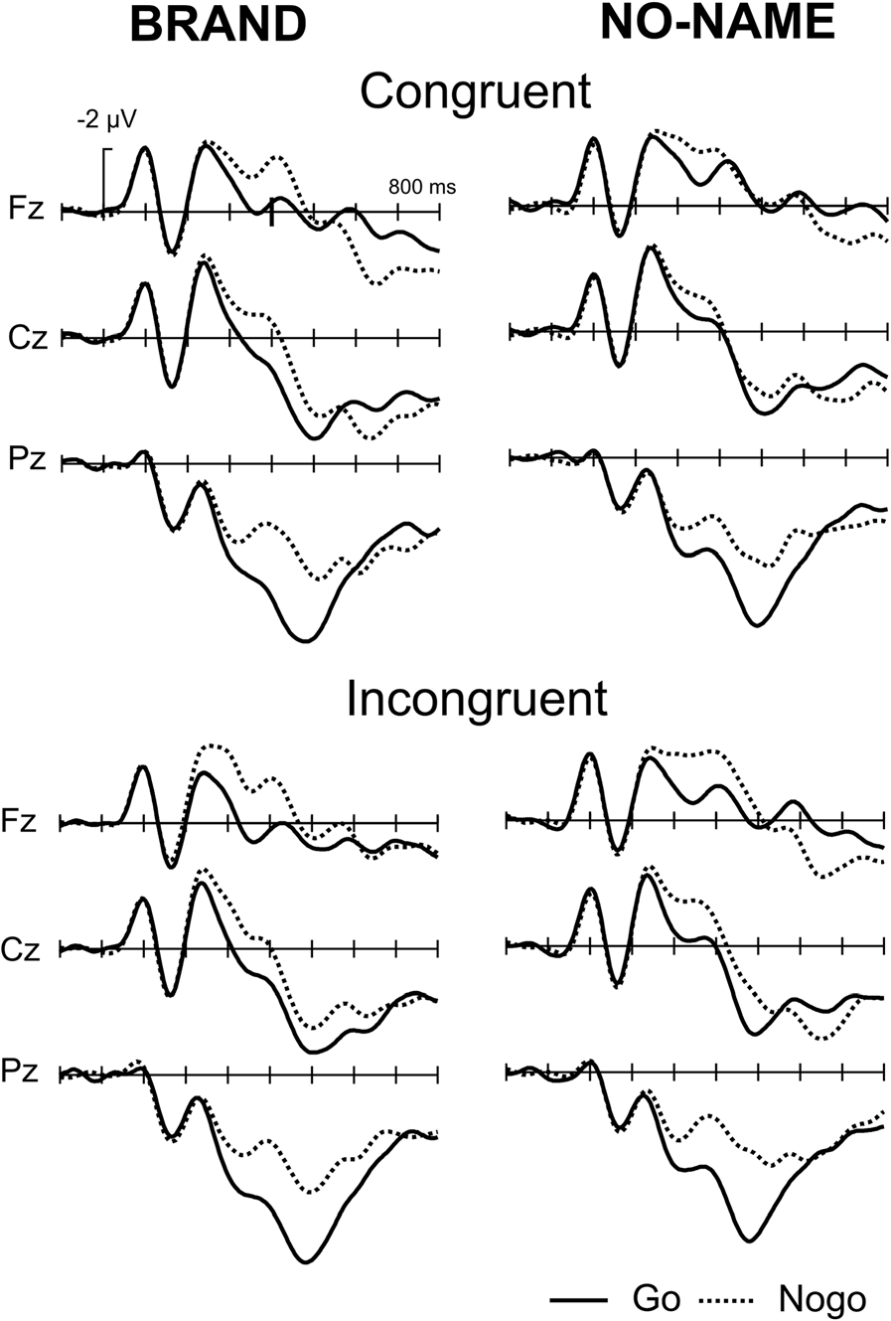
Grand Average ERPs, Go/Nogo effect: the ERPs for midline electrodes show a typical enhanced negativity for Nogo trials starting around 250 ms.

**Figure 3:**
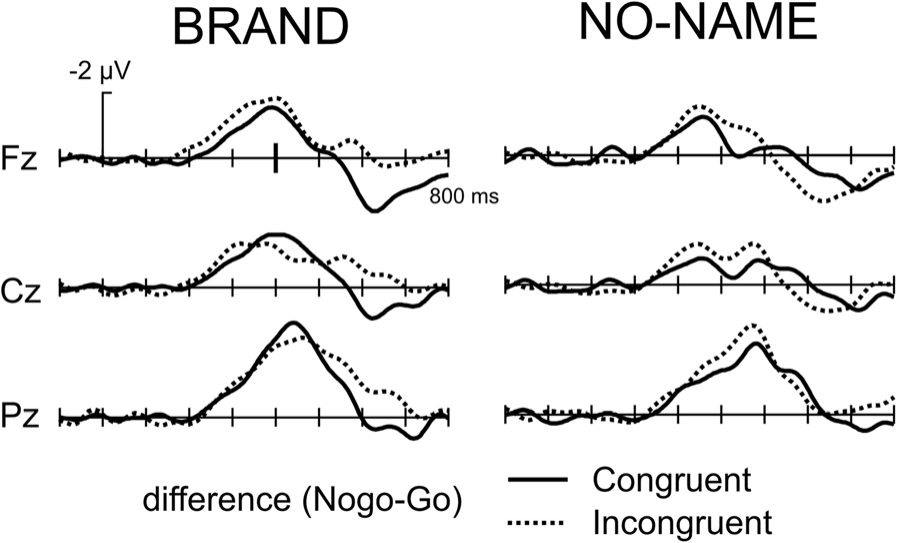
**Difference waves (Nogo-Go): difference waves show the typical phasic N200 response associated with Nogo trials.** There were no systematic latency differences as a function of congruency.

Figure 2 illustrates the basic Go/Nogo effect for brand and no-name stimuli. A typical N200 is present for brand and no-name stimuli for congruent and incongruent conditions. To isolate the N200, Nogo minus Go difference waves were computed (Figure 3). No systematic differences in latency of the N200 were seen as a function of congruency. Mean ERP amplitudes (200–400 ms, including central electrodes Fz, Cz, Pz) were subjected to a repeated measurement ANOVA revealing a highly significant main effect of Go/Nogo (F1,15) = 33.32, p < .001) but neither a main effect of Product (F(1,15) = 2.96) nor of Congruency (F(1,15) = 0.75). Also, interactions between the factor Go/Nogo and the other factors did not reach significance. The onset latency of the N200 was estimated by determining the point in time at which the negative area under the curve in the time window 200–400 ms reached 25% of its maximum. There was neither a main effect of Congruency (F(1,15) = 0.98) nor a Congruency by Product interaction (F(1,15) = 1.24).

**Figure 4:**
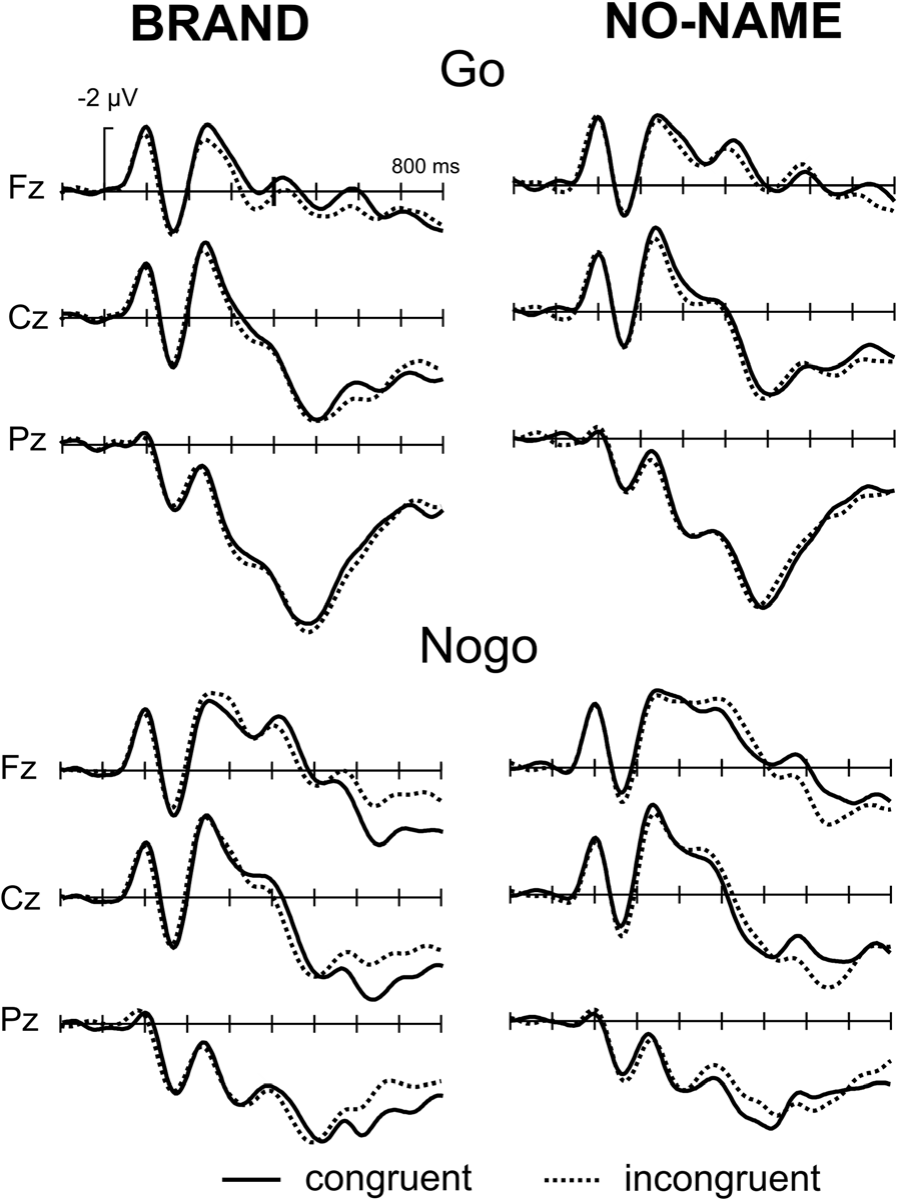
**Grand Average ERPs, Congruency effect: an effect of congruency is observed in the 600 – 800 ms time-window for the Nogo trials.** Incongruent stimuli are more negative in this time-window for the brand stimuli and more positive for the no-name stimuli.

Figure 4 illustrates the effect of congruency on the ERP. While there was no influence of congruency in the time-window of the N200 (see above), a later effect between 600–800 ms emerged in particular for the Nogo condition in that the congruent brand stimuli were associated with a less positive waveform. This effect was not seen for no-name products. This effect was quantified by a mean amplitude measure 600–750 ms. While the main effect of Congruency was not significant (F(1,15) = 0.87), we observed a triple interaction between Product x Go/Nogo x Congruency (F(1,15) = 4.97, p < 0.05) reflecting the fact that congruency had a differential influence in brand and no-name products and influenced only Nogo ERPs.

**Figure 5:**
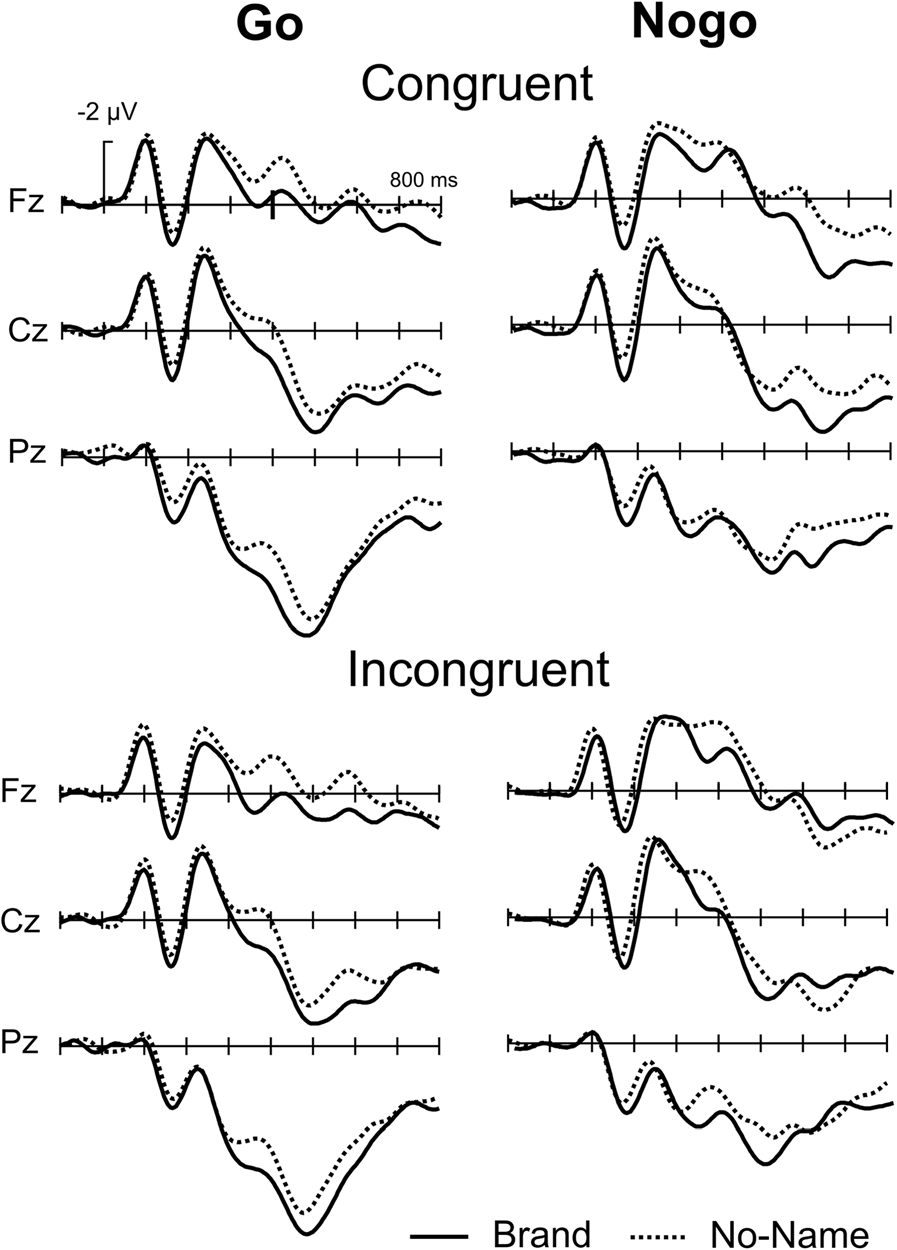
Grand Average ERPs, Brand effect: brand stimuli are associated with an enhanced late positive component compared to no-name products.

Figure 5 demonstrates the effects of brand status. Brand stimuli were associated with a more positive waveform compared to no-name products starting about 350 ms post stimulus onset and extending until about 700 ms. This effect was quantified by a mean amplitude measure in the 400 to 600 ms time window (P3/4, Pz, C3/4, Cz). The ANOVA revealed a main effect of Product (F(1,15) = 4.87, p < 0.05) and a main effect of Go/Nogo (F(1,15) = 15.1, p < 0.01) but neither an effect of Congruency (F(1,15) = 0.02) nor any interactions with that factor.

#### LRP

**Figure 6:**
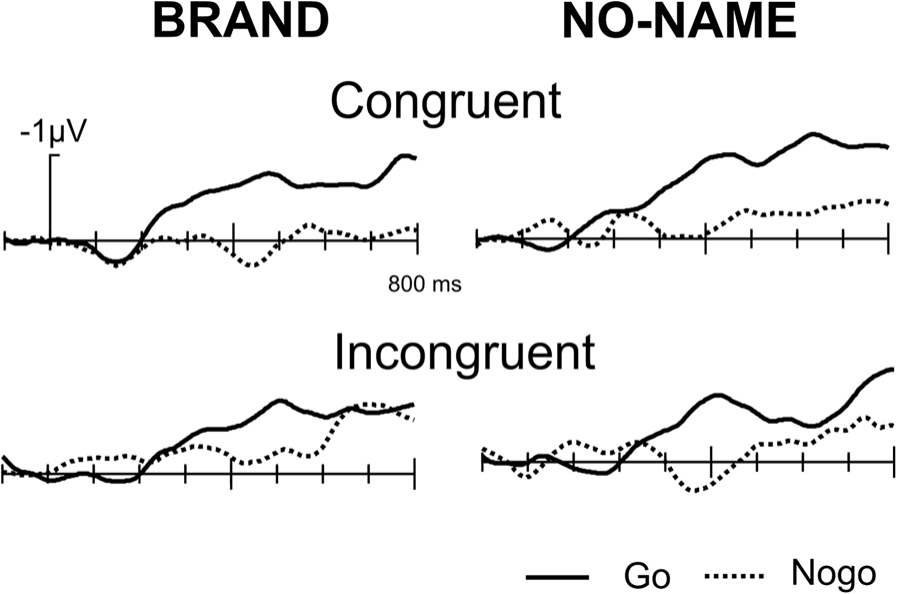
**Lateralized readiness potentials: a typical LRP is observed for the Go-trials.** No latency differences are present as a function of congruency. In the Nogo trials, no LRP emerges.

The grand average LRPs for brand and no-name products for congruent and incongruent conditions are shown in Figure 6. There were no apparent onset differences as a function of congruency for the Go-trials. The LRP was quantified by a mean amplitude measure in consecutive time-windows of 50 ms (starting at 100 ms post-stimulus). This analysis showed significant main effects for Go/Nogo between 300 and 500 ms. Moreover, a main effect was observed for the factor Product (F(1,15) = 5.95, p < .01) in the time-window 150 – 200 ms and a significant interaction of Product x Go/Nogo (F(1,15) = 4.57, p < .01) was seen in the window 350 ms to 400 ms. Other main effects or interactions failed to reach significance.

#### ERPs to words

**Figure 7:**
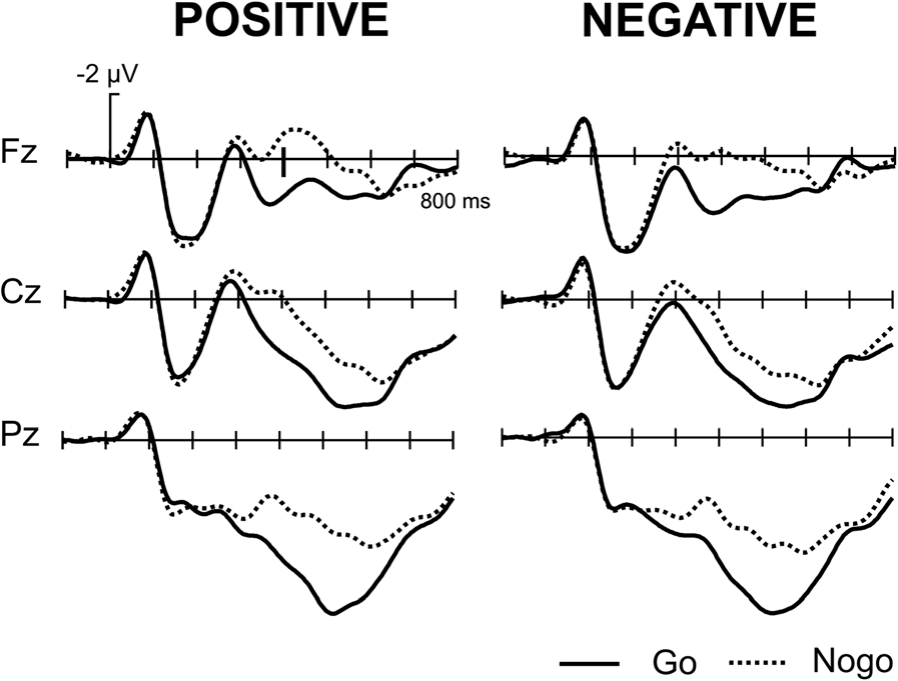
Grand Average ERPs, word stimuli: the ERPs to the word stimuli show a typical enhanced N200 component for the Nogo trials.

ERPs to words show a highly prominent effect of Go/Nogo (F(1,15) = 19.45, p < .001, time window 400 – 600 ms; Figure 7).

## Discussion

The present investigation was conducted to delineate the presence and time-course of implicit attitudes to brands and no-name products using the Go Nogo Association Task. We failed to observe a reaction time pattern indicative of implicit associations, which was puzzling as an unpublished fMRI experiment by our group using the same materials had revealed a robust RT effect. Also, we did not observe a latency difference of the N200 and LRP components as a function of congruency. Such latency differences had emerged in previous studies of our group investigating implicit attitudes towards “fruits and bugs” [26] and elderly and young faces [27]. However, we did observe later ERP differences as a function of congruency which occurred considerably later in time than the N200 component. Moreover, we also found a general difference between ERPs to brand and no-name products with the former having a more positive waveform starting around 300 ms.

### Implicit associations to brands?

As stated above, we failed to demonstrate the expected effects of congruency on response latencies to the brand and no-name product stimuli. It has to be pointed out, however, that the current experiment diverges from the standard procedure of the GNAT in several important ways: First, rather than presenting simple categories of items (e.g., fruits and insects) and pairing them with words of a specific valence, we used conjoint categories (brand cosmetics, no-name cosmetics, brand foods, no-name foods). Nosek and Banaji [18] have pointed out that conjoint categories might be special. For example, the evaluation of subgroups of larger social groups (e.g., race/ethnicity) may be different from that of the entire larger group. Thus, white Americans may be evaluated in a particular manner that is dissimilar to evaluations of Italian Americans and Polish Americans. In addition, in our experiment participants had to make judgements about class membership (cosmetics, food items) but we were interested in within class differences in implicit associations (e.g., brand cosmetics, no-name cosmetics).

In spite of these differences to standard GNAT designs, an fMRI experiment using the same stimulus materials (albeit with a different timing because of the requirements of the MRI-method) revealed a reaction time increase for incongruous relative to congruous stimuli of 50 ms. This was true for both, brands (ΔRT 25 ms) and no-name products (ΔRT 74 ms). Please note, that as in the current task the following pairings were considered congruous: brands + positive words, no-name products + negative words. At this point we can therefore only speculate as to why there was no effect of congruity in the present set of data? One possible explanation concerns the participants: Whereas the participants of the MRI study were 29 years old (mean), mostly working in academic professions and from Hannover, West Germany, the participants of the current EEG study were considerably younger (22 years), students and from Magdeburg, East Germany. It is conceivable that the difference in environment, age and financial resources might have influenced implicit attitudes towards brand and no-name food and cosmetic products.

In light of the missing difference for the response latencies for congruent and incongruent stimuli, it is no surprise that no latency differences were observed for the N200 component of the ERP or the LRP. There have been, however, congruity-related modulations of the ERP in a later time-window (600–800 ms) in particular for the Nogo condition. Interestingly, whereas for brand stimuli the incongruous condition led to a more negative waveform in this time-range, the opposite effect was observed for the no-name stimuli. A previous study, using a structurally similar Go/Nogo paradigm in the assessment of bilingual language processing also described such late effects of incongruity in Nogo trials (see Figure 3 in [46]). In this study incongruency effects for the go-responses were seen in the latency range of the N200 component and consequently was interpreted as reflecting partial inhibition of the go response. By contrast, an increased negativity due to incongruency with an onset latency of about 600 ms was seen for the nogo responses. This was seen as a reflection of inhibition as the particular stimulus contained information favoring a go-response. For the brand stimuli of the current study, a similar logic can be applied: Consider nogo brand stimuli in incongruent blocks: In these blocks positive words have to be responded to whereas (positively valued) brands are associated with a nogo response. The positive connotation should lead to a tendency to press the button which needs to be suppressed, manifesting itself in the late ERP effect.

### Differences between brands and no-name products

Interestingly, we observed general differences between brands and no-name products. First, responses to brand products were about 25 ms faster. Secondly, the ERPs to brand products were associated with a more positive waveform. In the ERP literature, a number of late positivities have been described, such as the P3/P300 [48,49] and the late positive component [50].

Whereas the P3 in visual tasks is usually rather peaked in appearance and occurs with latencies between 300 and 500 ms, the late positive components have a more extended waveshape and have been decribed in a number of situations, including retrieval of items from memory [51-53] but particularly in response to emotional stimuli. A long-lasting elevated ERP positivity to emotional, arousing pictures is a common finding [54-62]. It has been reported that pleasant as well as unpleasant stimuli elicit more positive-going ERPs in the 300–900 ms range and that such stimuli are recalled more often than neutral stimuli [61]. From its latency and distributional characteristics the current enhanced positivity for brand products could be related to these earlier findings, suggesting that brand products are more arousing which in turn might lead to a better retrieval and ultimately might consumers’ decisions.

LPC enhancement has also been reported for familiar compared to unfamiliar stimuli [63]. As the relative familiarity of brand products and no name products differed, an alternative explanation of the greater LPC could be that this simply reflects different degrees of familiarity rather than differential emotional engagement. At this point, we are unable to distinguish between these two possibilities.

## Conclusion

We did not observe a response latency difference or N200 latency difference between congruent and incongruent pairings of brand/no-name products and positive/negative words. Further research is needed to assess whether this was due to our specific adaptation of the GNAT-paradigm. The addition of ERPs to the GNAT has proven useful in the current experiment, as we could reveal late effects of congruency as well as a more general effect of brands vs. no-name products indicative of a deeper processing of brands. The IAT (e.g., [10,21,23,64] and priming paradigms [3] may present an additional fruitful avenue to explore the neural effects of implicit attitudes towards brands.

## Methods

All procedures and materials were approved by the institutional review board of the University of Magdeburg, Germany, the affiliation of the senior author at the time of the experiment.

### Participants

Sixteen young students of the University of Magdeburg (13 women, mean age 22.4 +/− 3.4) gave written consent to participate for financial compensation or course credit. All had normal or corrected-to-normal vision, were right-handed, neurologically healthy and native German speakers. Eight further participants were lost due to technical difficulties or excessive artefacts.

### Stimuli and procedure

An illustration of the basic classes of stimuli and the timing of the experiment is given in Figure 1. The critical stimuli were color photographs of brands (n = 24) and no-name products (n = 24). We made sure that we had pairs of stimuli (e.g., one famous type of coffee and one no-name coffee) and that the photographs of these pairs were matched as closely as possible for the angle from which the photo was taken, size of the product on the photo and background (all black). Also, the graphical complexity of the label was similar. Thus, no-name products with plain labels (“body lotion”, “yoghurt”) without graphical elaboration were avoided. Half of the pictures in each category (brands/no-name products) depicted cosmetics, the other half depicted food items, thus yielding 4 stimulus categories (cosmetic brand, food brand, cosmetic no-name, food no-name). Pictures were taken for the study and were presented in the center against a black background. The stimuli were assessed for familiarity in a further group of young healthy adults (n = 15, 8 women, age 21–35, mean age 28.1 years) on a 5 point Likert scale ranging from 1 (not at all familiar) to 5 (very familiar). The brand-name products had a high degree of familiarity (mean 4.3, SD 0.9), whereas the familiarity of the no-name products was lower (mean 2.7, SD 1.6; p < 0.01).

In addition, 48 word stimuli (half with a negative valence, e.g. bomb; half with a positive valence, e.g. sunshine) were taken from Banfield et al. [26] and were matched for length and frequency using the CELEX data base [65]. Stimuli were presented on a video-monitor using the software Presentation (Neurobehavioral Systems, Inc).

The experiment was subdivided into 8 runs of 5 minutes duration comprising the presentation of 96 trials each. One trial consisted of the presentation of the stimulus (duration of 500 ms) followed by a fixation cross (duration between 2000 and 3000 ms, rectangular distribution). In each run all 24 pictures of food, 24 pictures of cosmetic products, 24 positive words and 24 negative words (as “arrogant”) were presented in pseudo-randomized order with the condition that each stimulus category was not to be repeated more than twice in a row. Each of the eight runs featured a different instruction for the participant:

1. Press the button with the left hand for food products and positive words and withhold a response to cosmetics and negative words.

2. Press the button with the left hand for food products and negative words and withhold a response to cosmetics and positive words.

3. Press the button with the left hand for cosmetic products and positive words and withhold a response to food and negative words.

4. Press the button with the left hand for cosmetic products and negative words and withhold a response to food and positive words.

5.-8. As in 1 to 4 but with right hand button presses.

Please note that the participants were not aware that the main purpose of the study was to distinguish behavioural responses and brain activity to brands and no-name products. Responses were given using a modified computer mouse held either in the left or right hand. Statistical analysis of response latencies was carried out by analysis of variance (ANOVA) with product (brand, no name), congruency (congruent, incongruent) and response hand (left, right) as within subject factors.

The order of the runs/instructions was counterbalanced across participants. The whole experiment lasted approximately 43 minutes (excluding electrode application, instructions for the different runs, and debriefing). Participants were tested in a dimly lit room while sitting in a comfortable chair. The distance to the display was 80 cm.

### ERP recording and data analyses

The ERPs were recorded from the scalp using tin electrodes mounted in an elastic cap and located at 29 standard positions (Fpz, Fz, Cz, Pz, Fp1/2, F3/4, C3/4, P3/4, O1/2, F7/8, T3/4, T5/6, Fc1/2, Cp1/2, Fc5/6, Cp5/6). A reference electrode was placed on the left mastoid process. Vertical eye movements were monitored with an electrode at the infraorbital ridge of the right eye against Fpz (vertical EOG) and with a bipolar montage between two electrodes placed on the lateral canthi of the left and right eye (horizontal EOG). Electrode impedances were kept below 10 kOhm.

The electrophysiological signals were filtered with a bandpass of 0.01-70 Hz (half-amplitude cutoffs) and digitized at a rate of 250 Hz. Trials on which base-to-peak electro-oculogram (EOG) amplitude exceeded 200 μV, amplifier saturation occurred, or the baseline shift exceeded 250 μV/s were automatically rejected off-line. Datasets with more than 30% of the trials rejected were excluded from further analysis. For the remaining participants mean rejection rate was 16.2%.

Artifact free and correct trials were averaged separately for each stimulus type and condition over epochs of 1024 ms starting 100 ms prior to the stimulus. ERPs from the different conditions were later combined to yield 4 basic conditions. For all statistical effects involving two or more degrees of freedom in the numerator, the Greenhouse-Geisser epsilon was used to correct possible violations of the sphericity assumption. Exact p-value after correction will be reported. Tests involving electrode x condition interactions (e.g., factors as hemisphere or anterior-posterior electrode location) were carried out on data corrected using the vector normalization procedure described by McCarthy and Wood [66].

LRPs were assessed by using C3 and C4 electrode locations, where the amplitude of the readiness potential is maximum [28]. The LRP is computed by a double subtraction as shown in the following equation:

$$\mathrm{LRP}=\mathrm{left}\;\mathrm{hand}\;\left(C4–C3\right)–\mathrm{right}\;\mathrm{hand}\;\left(C4–C3\right)$$

Left and right hands refer to the expected correct hand and (C4 - C3) is the difference in electrical potential between these electrodes [29-31].

Mean amplitudes and peak latencies were computed for different time-windows, which were subjected to analyses of variance (ANOVA) with the factors Product (brand and no-name), Congruency (congruent and incongruent), Go/Nogo (Go and Nogo) and Electrode site.

## Competing interests

The authors declare that the research was conducted in the absence of any commercial or financial relationships that could be construed as a potential conflict of interests.

## Authors’ contributions

AT Performed the experiments and analyses, wrote first draft of the manuscript. AH co-designed the experiments, helped in the acquisition of the data and the statistical analysis. GB contributed to the design and revised the manuscript critically for important intellectual content. TFM conceived and designed the experiment and wrote the final version of the manuscript. All authors read and approved the final manuscript.

## References

[B1] Kotler P Keller KL Marketing Management 2009 New York: Prentice Hall International

[B2] Ajzen I Fishbein M The prediction of behavioral intentions in a choice situation J Exp Soc Psychol 1969 5 400 416

[B3] Mast FW Zaltman G A behavioral window on the mind of the market: an application of the response time paradigm Brain Res Bull 2005 67 422 427 16216689 10.1016/j.brainresbull.2005.06.004

[B4] Wicker AW Attitudes versus actions: the relationship of verbal and overt behavioral responses to attitude objects J Soc Issues 1969 25 41 78

[B5] Puccinelli NM Braun K Mast F Zaltman G Implicit predictors of consumer behavior 2001 Cambridge, Ma: Harvard Business School Publishing

[B6] Bargh JA Chen M Burrows L Automaticity of social behavior: direct effects of trait construct and stereotype activation on action J Pers Soc Psychol 1996 71 230 244 8765481 10.1037//0022-3514.71.2.230

[B7] Stanley D Phelps EA Banaji MR The neural basis of implicit attitudes Curr Dir Psychol Sci 2008 17 164 170

[B8] Adaval R How good gets better and bad gets worse: understanding the impact of affect on evaluations of known brands J Consum Res 2003 30 352 367

[B9] Dimofte CV Implicit measures of consumer cognition: a review Psychol Market 2010 27 921 937

[B10] Friese M Wänke M Plessner H Implicit consumer preferences and their influence on product choice Psychol Market 2006 23 727 740

[B11] Nevid JS Introduction to the special issue: implicit measures of consumer response-the search for the holy grail of marketing research Psychol Market 2010 27 913 920

[B12] Erk S Spitzer M Wunderlich AP Galley L Walter H Cultural objects modulate reward circuitry NeuroReport 2002 13 2499 2503 12499856 10.1097/00001756-200212200-00024

[B13] Schaefer M Rotte M Favorite brands as cultural objects modulate reward circuit NeuroReport 2007 18 141 145 17301679 10.1097/WNR.0b013e328010ac84

[B14] Schaefer M Rotte M Thinking on luxury or pragmatic brand products: brain responses to different categories of culturally based brands Brain Res 2007 1165 98 104 17655834 10.1016/j.brainres.2007.06.038

[B15] Schaefer M Rotte M Combining a semantic differential with fMRI to investigate brands as cultural symbols Soc Cogn Affect Neurosci 2010 5 274 281 20080877 10.1093/scan/nsp055 PMC2894682

[B16] Schaefer M Neuroeconomics: in search of the neural representation of brands Prog Brain Res 2009 178 241 252 19874974 10.1016/S0079-6123(09)17817-2

[B17] Kelley WM Macrae CN Wyland CL Caglar S Inati S Heatherton TF Finding the self? An event-related fMRI study J Cogn Neurosci 2002 14 785 794 12167262 10.1162/08989290260138672

[B18] Nosek BA Banaji MR The GO/NO-GO association task Soc Cogn 2001 19 625 664

[B19] Greenwald AG McGhee DE Schwartz JLK Measuring individual differences in implicit cognition: the implicit association test J Pers Soc Psychol 1998 74 1464 1480 9654756 10.1037//0022-3514.74.6.1464

[B20] Grover VP Keel PK Mitchell JP Gender differences in implicit weight identity Int J Eat Disord 2003 34 125 135 12772177 10.1002/eat.10167

[B21] Maison D Greenwald AG Bruin R Predictive validity of the implicit association test in studies of brands, consumer attitudes, and behavior J Consum Psychol 2004 14 405 415

[B22] Okubo S Ideno T Takemura K Implicit recognition test in studies on consumer behavior - possible application of implicit association test: IAT J Japan Res Assoc Textile End-Uses 2007 48 18 24

[B23] Perkins A Forehand M Greenwald AG Maison D Haugtvedt C, Herr P, Kardes F The influence of implicit social cognition on consumer behavior: measuring the non-conscious Handbook of Consumer Psychology 2008 Hillsdale, NJ: Lawrence Erlbaum Associates 461 475

[B24] Steinman RB Karpinski A The single category implicit association test (SC-IAT) as a measure of implicit consumer attitudes Eur J Soc Sci 2008 7 32 42

[B25] Beattie G Sale L Shopping to save the planet? Implicit rather than explicit attitudes predict low carbon footprint consumer choice Int J Environ Cult Econ Soc Sustainability 2011 7 211 232

[B26] Banfield JF van der Lugt AH Münte TF Juicy fruit and creepy crawlies: an electrophysiological study of the implicit Go/NoGo association task Neuroimage 2006 31 1841 1849 16581266 10.1016/j.neuroimage.2006.02.017

[B27] van der Lugt AH Banfield JF Osinsky R Münte TF Brain potentials show rapid activation of implicit attitudes towards young and old people Brain Res 2012 1429 98 105 22088825 10.1016/j.brainres.2011.10.032

[B28] Kutas M Donchin E Preparation to respond as manifested by movement-related brain potentials Brain Res 1980 202 95 115 7427748

[B29] Coles MGH Modern mind-brain reading: psychophysiology, physiology and cognition Psychophysiology 1989 26 251 269 2667018 10.1111/j.1469-8986.1989.tb01916.x

[B30] Gratton G Coles MGH Sirevaag EJ Eriksen CW Donchin E Pre- and poststimulus activation of response channels: a psychophysiological analysis J Exp Psychol Hum Percept Perform 1988 14 331 344 2971764 10.1037//0096-1523.14.3.331

[B31] Smid HGOM Mulder G Mulder LJM Brands GJ A psychophysiological study of the use of partial information in stimulus–response translation J Exp Psychol Hum Percept Perform 1992 18 1101 1119 1431747 10.1037//0096-1523.18.4.1101

[B32] Miller J Schaffer R Hackley SA Effects of preliminary information in a Go versus No-go task Acta Psychol 1991 76 241 292 10.1016/0001-6918(91)90022-r 1927576

[B33] Schmitt BM Münte TF Kutas M Electrophysiological estimates of the time course of semantic and phonological encoding during implicit picture naming Psychophysiology 2000 37 473 484 10934906

[B34] Schmitt BM Schiltz K Zaake W Kutas M Münte TF An electrophysiological analysis of the time course of conceptual and syntactic encoding during tacit picture naming J Cogn Neurosci 2001 13 510 522 11388923 10.1162/08989290152001925

[B35] van Turennout M Hagoort P Brown C The time course of grammatical and phonological processing during speaking: evidence from event-related brain potentials J Psycholinguist Res 1999 28 649 676 10510863 10.1023/a:1023221028150

[B36] van Turennout M Hagoort P Brown C Electrophysiological evidence on the time course of semantic and phonological processes in speech production J Exp Psychol Learn Mem Cogn 1997 23 787 806 9445890 10.1037//0278-7393.23.4.787

[B37] Gemba H Sasaki K Potential related to no-go reaction of go/no-go hand movement task with color discrimination in human Neurosci Lett 1989 101 263 268 2771172 10.1016/0304-3940(89)90543-0

[B38] Pfefferbaum A Ford JM Weller BJ Kopell BS ERPs to response production and inhibition Electroenceph Clin Neurophysiol 1985 60 423 434 2580694 10.1016/0013-4694(85)91017-x

[B39] Sasaki K Gemba H Electrical activity in the prefrontal cortex specific to no-go reaction of conditioned hand movement with colour discrimination in the monkey Exp Brain Res 1986 64 603 606 3803495 10.1007/BF00340499

[B40] Simson R Vaughan J Ritter W The scalp topography of potentials in auditory and visual Go/Nogo tasks Electroenceph Clin Neurophysiol 1977 43 864 875 73454 10.1016/0013-4694(77)90009-8

[B41] Sasaki K Gemba H Tsujimoto T Suppression of visually initiated hand movement by stimulation of the prefrontal cortex in the monkey Brain Res 1989 495 100 107 2776028 10.1016/0006-8993(89)91222-5

[B42] Aron AR Robbins TW Poldrack RA Inhibition and the right inferior frontal cortex Trends Cogn Sci 2004 8 170 177 15050513 10.1016/j.tics.2004.02.010

[B43] Garavan H Ross TJ Stein EA Right hemispheric dominance of inhibitory control: an event-related functional MRI study Proc Natl Acad Sci U S A 1999 96 8301 8306 10393989 10.1073/pnas.96.14.8301 PMC22229

[B44] Wager TD Sylvester CYC Lacey SC Nee DE Franklin M Jonides J Common and unique components of response inhibition revealed by fMRI Neuroimage 2005 27 323 340 16019232 10.1016/j.neuroimage.2005.01.054

[B45] Rodriguez-Fornells A Schmitt BM Kutas M Münte TF Electrophysiological estimates of the time course of semantic and phonological encoding during listening and naming Neuropsychologia 2002 40 778 787 11900728 10.1016/s0028-3932(01)00188-9

[B46] Rodriguez-Fornells A van Der Lugt A Rotte M Britti B Heinze HJ Münte TF Second language interferes with word production in fluent bilinguals: brain potential and functional imaging evidence J Cogn Neurosci 2005 17 422 433 15814002 10.1162/0898929053279559

[B47] McClure SM Li J Tomlin D Cypert KS Montague LM Montague PR Neural correlates of behavioral preference for culturally familiar drinks Neuron 2004 44 379 387 15473974 10.1016/j.neuron.2004.09.019

[B48] Münte TF Urbach TP Düzel E Kutas M Event-related brain potentials in the study of human cognition and neuropsychology Handb Neuropsychol 2000 1 139 235

[B49] Polich J Updating P300: an integrative theory of P3a and P3b Clin Neurophysiol 2007 118 2128 2148 17573239 10.1016/j.clinph.2007.04.019 PMC2715154

[B50] Olofsson JK Nordin S Sequeira H Polich J Affective picture processing: an integrative review of ERP findings Biol Psychol 2008 77 247 265 18164800 10.1016/j.biopsycho.2007.11.006 PMC2443061

[B51] Azizian A Polich J Evidence for attentional gradient in the serial position memory curve from ERPs J Cogn Neurosci 2007 19 2071 2081 17892393 10.1162/jocn.2007.19.12.2071 PMC2748728

[B52] Karis D Fabiani M Donchin E P300 and memory—individual differences in the von Restorff effect Cogn Psychol 1984 16 177 216

[B53] Paller KA McCarthy G Wood CC ERPs predictive of subsequent recall and recognition performance Biol Psychol 1988 26 269 276 3207786 10.1016/0301-0511(88)90023-3

[B54] Amrhein C Muhlberger A Pauli P Wiedemann G Modulation of event-related brain potentials during affective picture processing: a com-plement to startle reflex and skin conductance response? Int J Psychophysiol 2004 54 231 240 15331214 10.1016/j.ijpsycho.2004.05.009

[B55] Cuthbert BN Schupp HT Bradley MM Birbaumer N Lang PJ Brain potentials in affective picture processing: covariation with autonomic arousal and affective report Biol Psychol 2000 52 95 111 10699350 10.1016/s0301-0511(99)00044-7

[B56] Ito T Cacioppo JT Lang PJ Eliciting affect using the international affective picture system: trajectories through evaluative space Pers Soc Psychol Bull 1998 24 855 879

[B57] Ito TA Larsen JT Smith NK Cacioppo JT Negative information weighs more heavily on the brain: the negativity bias in evaluative categorizations J Pers Soc Psychol 1998 75 887 900 9825526 10.1037//0022-3514.75.4.887

[B58] Keil A Muller MM Gruber T Wienbruch C Stolarova M Elbert T Effects of emotional arousal in the cerebral hemispheres: a study of oscillatory brain activity and event-related potentials Clin Neurophysiol 2001 112 2057 2068 11682344 10.1016/s1388-2457(01)00654-x

[B59] Mini A Palomba D Angrilli A Bravi S Emotional information processing and visual evoked brain potentials Percept Mot Skills 1996 83 143 152 8873187 10.2466/pms.1996.83.1.143

[B60] Olofsson JK Polich J Affective visual event-related potentials: arousal, repetition, and time-on-task Biol Psychol 2007 75 101 108 17275979 10.1016/j.biopsycho.2006.12.006 PMC1885422

[B61] Palomba D Angrilli A Mini A Visual evoked potentials, heart rate responses and memory to emotional pictorial stimuli Int J Psychophysiol 1997 27 55 67 9161892 10.1016/s0167-8760(97)00751-4

[B62] Schupp HT Cuthbert BN Bradley MM Cacioppo JT Ito T Lang PJ Affective picture processing: the late positive potential is modulated by motivational relevance Psychophysiology 2000 37 257 261 10731776

[B63] Hou M Safron A Paller KA Guo C Neural correlates of familiarity and conceptual fluency in a recognition test with ancient pictographic characters Brain Res 2013 1518 48 60 23632379 10.1016/j.brainres.2013.04.041

[B64] Maison D Greenwald AG Bruin R The implicit association test as a measure of implicit consumer attitudes Pol Psychol Bull 2001 2 61 79

[B65] Baayen RH Piepenbrock R Van Rijn H The CELEX lexical database [CD-ROM] 1993 Philadelphia: Linguistic Data Consortium, University of Pennsylvania

[B66] McCarthy G Wood CC Scalp distributions of event-related potentials: an ambiguity associated with analysis of variance models Electroenceph Clin Neurophysiol 1985 62 203 208 2581760 10.1016/0168-5597(85)90015-2

